# Improving the Conduct of Health-Related Research in Schools

**DOI:** 10.3389/phrs.2025.1608316

**Published:** 2025-08-21

**Authors:** Alessia Raineri, Manuel Weber, Seraina Rüegger, Susi Kriemler, Milo A. Puhan

**Affiliations:** ^1^ Epidemiology, Biostatistics and Prevention Institute, University of Zurich, Zurich, Switzerland; ^2^ Swiss School of Public Health, Zurich, Switzerland; ^3^ Academic-Practice-Partnership Between the School of Health Professions at Bern University of Applied Sciences and the University Hospital of Bern, Bern University of Applied Sciences, Bern, Switzerland; ^4^ Population Research Center, University of Zurich, Zurich, Switzerland

**Keywords:** children and adolescents, collaboration, incentive, motivation, partnership, prevention, recruitment, retention

## Abstract

**Background:**

Schools serve not only as centers of education but also as critical environments for social development and promoting health and well-being of children and adolescents. Conducting health-related research in school settings offers valuable opportunities to identify current health challenges and assess preventive and interventional strategies.

**Analysis:**

Researchers face several challenges when conducting health-related research in schools, including communication barriers, complex coordination among relevant stakeholders, and limited resources.

**Policy Options:**

This policy brief, with a special focus on Switzerland, highlighted main challenges and presented options derived from literature, semi-structured interviews, and a stakeholder dialogue to address them. The dialogue included representatives from youth, parents, teachers, school principals, school authorities, and researchers. The results emphasize key options in three main areas: (i) recruitment and motivation, (ii) retention and incentives, and (iii) partnerships and collaboration.

**Conclusion:**

Many of the proposed options do not require large-scale political shifts nor national-level policy changes but can directly be applied in schools and research settings, emphasizing their practical applicability and potential for immediate impact. Ultimately, implementing these options in health-related research in schools can contribute to long-term health and well-being of future generations.

## Background

The future of our society begins in the classroom. Not only do schools play a central role in the transmission of knowledge, but they also serve as key environments for understanding and promoting the health and well-being of children and adolescents [1]. Therefore, health-related research in schools (HRRS) is important for identifying current and future health issues and developing and evaluating preventive measures as well as cost-effective health programs. Schools provide unique settings for scientific research, as children and adolescents spend a substantial part of their daily lives there. The socioeconomic diversity within schools, encompassing factors such as parental education levels and household income, allows for the collection of more comprehensive data compared to specialized environments, such as sports or youth clubs [2]. Often, schools are the only option for studies that aim to draw conclusions about the general population of children and adolescents.

Research in school settings is critical for generating insights that support UNICEF’s and the UN’s third Sustainable Development Goal: SDG 3 – Good Health and Well-Being [3, 4], particularly sub-goal 3.4, which focuses on reducing mortality from non-communicable diseases and promoting mental health and well-being. HRRS enables a deeper understanding of the physical, mental, and social dimensions of health among children and adolescents, providing essential evidence to inform targeted interventions and health-promoting policies. This comprehensive approach is essential for developing effective strategies to promote the health and well-being of children and adolescents. It supports the early identification of health risks and the analysis of interrelated factors, which in turn inform the design of interventions, such as school-based health programs for children and adolescents [2, 5]. Ultimately, these efforts could contribute to long-term, sustainable improvements in their overall quality of life.

In 2019, an estimated 7%–14% of children and adolescents aged 5–19 years were affected by mental disorders [6], and clinical treatment for these disorders among children and adolescents has increased in recent decades [7]. In Switzerland, many teenagers face mental health challenges [8, 9] and report physical symptoms such as headaches, stomachaches, and back pain [10]. Furthermore, the majority of adolescents worldwide fail to meet recommended physical activity guidelines [11], a finding also reflected in a Swiss national study from 2019 [12]. Finally, the increasing global use of digital media introduces further health risks as higher levels of screen time are associated with reduced physical and psychosocial health among children and adolescents [13–15].

The Swiss context for HRRS is characterized by a unique blend of federalism, linguistic diversity, and a multicultural student population, alongside a dense network of public schools, with a limited number of private institutions. The federal structure distributes educational responsibilities among cantons, municipalities, and schools, which complicates the implementation of uniform health interventions. Switzerland’s educational system is highly decentralized, with 26 different scholastic systems coexisting within the same territory [16]. Such decentralization can complicate survey design and implementation, as researchers must navigate varying educational policies and structures across different cantons. Consequently, researchers often face challenges in identifying the appropriate stakeholders with whom to discuss their study plans. Additionally, many students and their families may not be native speakers of the local language, which creates barriers to effective communication and engagement in health initiatives [17]. With one of the highest proportions of foreign nationals in Europe, Swiss schools embody a rich tapestry of cultural backgrounds, presenting both challenges and opportunities for HRRS. This linguistic diversity necessitates tailored research approaches that accommodate multiple languages and cultural perspectives to ensure inclusivity and understanding [18].

Despite the need for HRRS, there are considerable challenges to overcome when conducting it. To the best of our knowledge, this is the first policy brief addressing how to improve the conduct of HRRS. First, we briefly summarized evidence of the challenges of conducting HRRS, with a particular focus on the Swiss context. Second, we outlined policy options across three main areas relevant to HRRS. Finally, we discussed potential barriers to and facilitators of the implementation of these policy options.

## Analysis

The National Health Report 2020 by the Swiss Health Observatory [19] presents six key recommendations, one of which emphasizes the need for the need for continuous data collection on children, adolescents, and young adults. Regular health data not only aids in the monitoring of health factors and outcomes but also allows for detailed analyses of their associations with learning outcomes and social determinants. Alongside routine data collection, targeted studies can address existing data gaps. Investing in the health and well-being of children and adolescents can yield significant benefits: for the present, in later adult life, and for future generations [1, 20]. Moreover, investments in children’s health can lead to reduced healthcare costs in the future by decreasing the prevalence of health problems [21, 22]. Healthy children are more likely to become healthy adults [19], which in turn brings long-term economic benefits to society [23]. For instance, childhood and adolescent overweight increases the risk of obesity and associated comorbidities in adulthood [24, 25].

Conducting HRRS is essential to understanding and addressing the future health issues faced by children and adolescents. Despite its importance, there is limited evidence on the most effective methods to conduct HRRS. However, the literature identifies several key challenges for relevant stakeholders (i.e., youth, parents, teachers, school principals, school authorities, and researchers) that hinder the successful execution of HRRS, as outlined in Table 1 [2, 5, 26–30].

**TABLE 1:** Challenges in health-related research in schools (Switzerland, 2025).

Challenges	Descriptions
Communication and understanding [2, 5, 27, 28, 30]	• Lack of exchange • Failure to use target group-specific communication • Different ideas and knowledge about study objectives and implementation
Complexity [2, 5, 26–30]	• Time-intensive coordination • Gaining access to schools and gatekeeping • Isolated and uncoordinated structures
Processes and guidelines [2, 5, 26, 28, 29]	• Partially cumbersome processes between the school and administrative bodies • Lack of clear and standardized guidelines for schools, teachers, and researchers • Lack of predefined processes
Resources [2, 5, 26–28]	• Time and financial constraints • Overburdening of school principals, teachers, and researchers • Suboptimal infrastructure
Incentives [2, 5, 26–28]	• Lack of motivation due to missing benefits • Lack of recognition and appreciation for participants • No win-win situations

Addressing these challenges requires a coordinated effort among all stakeholders, aligned with public engagement. Public engagement refers to actively and continuously involving the public and relevant stakeholders in scientific research [31]. To ensure the successful implementation of HRRS, clear communication strategies, simplified administrative processes, standardized guidelines, adequate resource allocation, and effective incentives must be established.

## Policy Options

**FIGURE 1:**
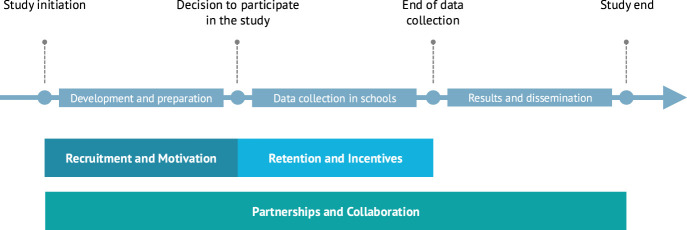
Temporal assignment of the three main areas of policy options in health-related research in schools (Switzerland, 2025).

Between January and March 2024, we conducted a rapid literature review and five semi-structured interviews with HRRS experts (each approximately 90 min), focusing on four central themes: implementation, facilitators, challenges, and experiences related to HRRS. Building on these findings, we organized a full-day stakeholder dialogue in May 2024, involving 13 representatives from various groups, including youth, parents, teachers, school principals, school authorities, and researchers. The dialogue served to expand the discussion of the four central themes mentioned above while gathering stakeholders’ perspectives. Drawing from the synthesized insights, this policy brief identified three main areas for targeted policy options aimed at effectively conducting HRRS. These areas are *recruitment and motivation*, *retention and incentives*, and *partnerships and collaboration,* and can be assigned within a study timeline as follows (Figure 1):

• Recruitment and motivation: This area mainly encompasses the period from the study’s initiation to the school’s decision to participate.
• Retention and incentives: This phase primarily covers the time from the school’s decision to participate until the completion of data collection.
• Partnerships and collaboration: This area involves all stages of the study.

The policy options within these main areas are designed to complement each other, allowing for simultaneous or sequential implementation. However, it should be acknowledged that our proposed policy options are not exhaustive and may not fully address all challenges associated with HRRS.

This policy brief is primarily targeted at individuals directly involved in the planning and execution of HRRS. These include students, parents, teachers, school principals, school authorities, and researchers – key stakeholders who are well-positioned to implement these options at the local level. Many of these suggestions do not require large-scale political shifts or national-level policy changes but can be directly applied in schools and research settings, emphasizing their practical applicability and potential for immediate impact.

### Options: Recruitment and Motivation

Recruiting and sustaining the motivation of participants are pivotal to the success of HRRS. Therefore, it is essential to identify gatekeepers and develop targeted strategies that effectively engage all stakeholders involved [32]. By understanding what motivates different stakeholders, researchers can enhance participation from the outset. Highlighting the potential benefits of participation is key [2, 33]. These benefits include personal insights into one’s health and contributions to the well-being of the broader community. Furthermore, it is important to emphasize both the short- and long-term benefits, including how HRRS could help schools better manage health issues over time.

Minimizing the effort and time commitment required of all parties involved, including authorities, is vital for successful implementation. Researchers should acknowledge the diverse needs of stakeholders and use tailored communication strategies to build trust and engagement. Transparent communication about data protection, security, workload, and timelines is essential. Offering materials in multiple languages and incorporating engaging formats, such as short video clips, can enhance accessibility.

For example, when approaching a primary school for a study on physical activity, researchers usually initiate contact from the top down through the school principal, who is often the key gatekeeper [34]. If the school is receptive, an online meeting can be organized with school staff to present the study. To engage students and parents, an informational event can be held, with school staff collaborating to ensure the clear and relatable communication of the study’s goals and benefits. Simple, concise materials and opportunities for further inquiry (such as online meetings or a hotline) can help clarify concerns and boost motivation to participate.

### Options: Incentives and Retention

Retaining the commitment and motivation of school principals, teachers, parents, and students is critical for the success of HRRS [2, 35]. One key strategy is to create regular opportunities for engagement with the researchers. Building trust through consistent communication and providing insights into the study’s progress helps maintain interest and recognize contributions [36]. In longitudinal studies, it is crucial to highlight the importance of participating in all phases of data collection, as this directly impacts the quality of the study. Researchers should offer flexible scheduling options and limit mandatory sessions to reduce strain on participants.

Consistency in communication is equally important. Assigning specific, easily identifiable contacts throughout the study, rather than generic email addresses, helps build and maintain trust and ensures participants know who to reach out to. Additionally, offering meaningful incentives, such as school trips or vouchers, can sustain motivation. Another powerful incentive is giving schools a voice in how the study is implemented, fostering a sense of partnership and ownership in addition to ensuring that the research is relevant to their needs.

For example, a secondary school participating in a study on adolescent mental health could stay engaged through regular feedback channels such as online surveys, virtual meetings, and newsletters. Providing students with individual, non-evaluative health reports could also foster participation. Furthermore, allowing the school to use study data to improve educational practices would ensure that the research benefits both students and school staff. By implementing these strategies, schools, parents, and students are more likely to stay committed, motivated, and engaged throughout the study, enhancing their success and impact.

### Options: Partnerships and Collaboration

Building strong, effective collaborations with all involved stakeholders is essential for the success of HRRS [2, 29]. Efficient cooperation with key authorities, such as ethics committees and school boards, is crucial from the outset. Open communication and a shared understanding among all parties help ensure that the study progresses smoothly. Providing practical resources, such as checklists and step-by-step guides, can streamline processes at both the administrative and school levels. Collaborating with other research institutions allows for the pooling of resources and expertise, which improves efficiency and reduces the time commitment required from participating schools. Learning from the best practices of previous studies, either initiated by policymakers or other researchers, further enhances the execution of the study.

At the school level, offering regular platforms for dialogue supports collaboration. Involving schools, parents, and students from the beginning ensures that their viewpoints and needs are addressed early on. This participatory approach prevents the perception of a “one-sided” study and fosters a genuine partnership [37]. Whenever possible, and depending on resources, schools, parents, and students can be directly involved in shaping the study itself [38]. Forming an advisory board with representatives from all groups increases transparency, builds trust, and enhances long-term commitment.

However, it is important to approach early involvement with care. Schools should not feel overwhelmed by the demands of participating in research. The initial contact should present a clear and carefully considered plan that is also flexible enough to allow for adjustments based on the school’s feedback. Schools can also help prioritize research topics and, when appropriate, integrate studies into their curriculum, thereby embedding the research further into everyday school life.

For example, in a study on blood biomarkers at primary schools, researchers could start by contacting school principals or relevant authorities regarding resources (e.g., ethics forms and recruitment processes). Once approval is granted, schools can activate internal communication channels. Researchers can offer interactive materials (e.g., videos and handouts) to engage students in an accessible way. This collaboration not only facilitates the research process but also enriches the students’ learning experience.

## Conclusion

HRRS is essential, as schools provide a unique environment for investigating the health of children and adolescents. Not only do schools allow for the identification of current health issues, but they also provide an opportunity to develop and assess preventive and interventional measures. However, conducting HRRS involves certain challenges. In this policy brief, we highlighted key challenges and offered targeted options in three main areas – recruitment and motivation, incentives and retention, and partnerships and collaboration. While our focus is primarily on Switzerland, these insights and options may also be relevant to other countries. By implementing these option, we can improve HRRS, ultimately contributing to the long-term health and well-being of future generations.

## ORIGINAL POLICY BRIEF

The original policy brief is available in German on the Swiss Learning Health System website (https://www.slhs.ch/en/policy-briefs-stakeholder-dialogues/our-topics/health-related-studies-in-schools-a-unique-opportunity/), along with the summary of the stakeholder dialogue, which took place on 21 May 2024, when 13 stakeholders representing youth, parents, teachers, school principals, school authorities and researchers discussed the policy brief. Subsequently, the policy brief was adapted according to the results of the stakeholder dialogue.
